# Factors Associated With Non-invasive Oxygen Therapy Failure in COVID-19 Pneumonia: A Single Center, Retrospective Study in a Tertiary Hospital in North India

**DOI:** 10.7759/cureus.29721

**Published:** 2022-09-28

**Authors:** Sekar L, Inderpaul S Sehgal, Kamal Kajal, Sandeep Kataria, Madhumita Premkumar, Karan Singla, Varun Mahajan, Deep K Gorla, Goverdhan D Puri

**Affiliations:** 1 Anesthesia and Intensive Care, Postgraduate Institute of Medical Education and Research, Chandigarh, IND; 2 Anesthesiology, All India Institute of Medical Sciences, Kalyani, Kalyani, IND; 3 Pulmonary Medicine, Postgraduate Institute of Medical Education and Research, Chandigarh, IND; 4 Anesthesiologyt, BronxCare Health System, Bronx, USA; 5 Hepatology, Postgraduate Institute of Medical Education and Research, Chandigarh, IND

**Keywords:** continuous positive airway pressure (cpap), high flow nasal cannula (hfnc), covid induced ards, rox index, predictors of nit failure, invasive ventilation, non-invasive oxygen therapy, non-invasive ventilation, covid-19

## Abstract

Background: Non-invasive oxygen therapy (NIT) consists of high-flow nasal oxygen (HFNO) and continuous positive airway pressure (CPAP). NIT is routinely being used for the management of acute respiratory failure secondary to coronavirus disease-2019 (COVID-19) with variable outcomes. However, previously published studies show that NIT failure might delay endotracheal intubation and invasive mechanical ventilation and results in worse outcomes in patients with hypoxemic respiratory failure. Early prediction of failure of NIT, will help in early decision-making in initiating invasive mechanical ventilation. We retrospectively studied the predictors for NIT failure in patients with moderate to severe COVID-19.

Methods: Adult patients (>18 years) admitted to the intensive care unit (ICU) with moderate to severe COVID-19 ARDS and received NIT [HFNO and CPAP non-invasive ventilation (NIV)] were included in this study. Baseline clinical and laboratory data were collected retrospectively from the electronic hospital information system. NIT failure was defined as the need for invasive mechanical ventilation after the initiation of NIT in the ICU. Univariate and multivariate logistic regression analyses were used to find out the possible predictors of NIT failure.

Results: Out of 254 patients admitted to ICU, 127 patients were initiated NIT at admission to ICU. During the course of the ICU stay, 33 (26%) patients subsequently required invasive mechanical ventilation (NIT failure). Respiratory rate-oxygenation index (ROX index) of <2.97 at two hours and <3.63 at six hours of ICU admission predicted NIT failure in our cohort of patients with a high positive predictive value.

Conclusion: Patient selection is crucial for successful NIT in COVID-19. Application of ROX index measured in the first six hours of ICU admission helps in the identification of patients at risk of NIT failure with moderate to severe COVID-19 ARDS.

## Introduction

Non-invasive high flow oxygen therapy (NIT) that includes high-flow nasal oxygen (HFNO) and non-invasive continuous positive airway pressure ventilation (NIV CPAP) has been successfully used for the management of acute hypoxemic respiratory failure due to coronavirus disease 2019 (COVID-19) [1-4]. However, non-judicious use of NIT may cause poor outcomes due to delayed initiation of invasive mechanical ventilation [5]. Apart from poor patient compliance, prolonged use of CPAP therapy results in facial injury, and alteration in the lung mechanics resulting in barotrauma probably secondary to large tidal volumes delivered and by the injurious transpulmonary pressures [6]. Early prediction of failure of NIT, might help in early decision-making in initiating invasive mechanical ventilation. This will also help in the wise allocation of available resources in resource-poor settings.

In this study, we retrospectively studied the possible predictive factors associated with NIT failure in adult patients with moderate to severe COVID-19 pneumonia admitted to the intensive care unit (ICU) in a tertiary care hospital in North India.

## Materials and methods

Study design and setting

This single-center, retrospective, observational study was conducted in the Postgraduate Institute of Medical Education and Research, Chandigarh, India. All consecutive adult patients (>18 years) of either sex were admitted to the dedicated COVID-19 ICU between May 2020 and January 2021 with moderate to severe COVID-19 ARDS (Adult Respiratory Distress Syndrome) (PaO_^2^_/FiO_2_) and received NIT in the form of HFNO (AIRVO_2_, Fischer & Paykel Healthcare) or NIV CPAP (Hamilton C3S, Hamilton Medical Inc. USA) were included in this study. The protocol for the selection of oxygen therapy is shown in Appendix. The flow and the fractional inspired oxygen concentration in HFNO were adjusted according to respiratory rate and oxygen saturation in the pulse oximeter respectively. Pressure support settings in NIV CPAP were adjusted to the tidal volume of 6 mL kg^-1^. Patients, who received invasive ventilation before the institution of NIT were excluded. Patients who received NIT, subsequently improved, and were discharged from the ICU were considered as “NIT success”. Those who received invasive mechanical ventilation after the institution of NIT due to progression of disease severity during the ICU stay were considered as “NIT failure”. All patients received standard medical therapy consisting of steroids, therapeutic anticoagulation, antiviral drugs, and organ-specific supportive therapy according to the latest evidence available at that time.

Data collection

Patient demographic details (age, sex, body mass index [BMI]) and laboratory parameters (hemogram, biochemistry, coagulation tests, and inflammatory markers) were collected retrospectively from the electronic hospital information system. Written medical records were used inside the ICU whereas digital medical records were maintained for the purpose of e-rounds for the management discussion by the multidisciplinary team. Data were accessed retrospectively from the digital health records (co-morbid conditions, symptom onset and progression, order of symptom appearance, vitals during the ICU stay, sequential organ failure assessment [SOFA] score upon arrival to the ICU, oxygen requirements, blood gas analysis, drug therapy, and ventilation strategy) while maintaining the privacy and confidentiality. Data were available till the discharge of the patient from the ICU. The duration of NIT and the need for invasive ventilation after the initial NIT were also noted down.

Statistical analysis

Continuous variables were presented as the median with interquartile range (IQR); Categorical variables were reported as percentages and compared using the Chi-square test. Fisher’s exact test/Chi-square test was also used to estimate continuous variables. The Mann-Whitney U-test was used to study non-parametric continuous variables. All variables associated with the NIT failure in the univariate model with p < 0.05 were entered into a backward-logistic multivariate regression model and the odds ratio (OR) with 95% confidence intervals (95% CI) was calculated. A receiver operating characteristic (ROC) curve was constructed to display the area under the curve (AUC) for the predictive model. The optimal cut-off was considered as the one showing the best accuracy using Youden's J statistic. At this cut-off value, the performance of the model is presented as sensitivity, specificity, and positive and negative predictive values. A value of p < 0.05 was considered statistically significant. The statistical analysis was conducted using IBM SPSS Statistics version 24.0 software (IBM Corp., Armonk, NY, USA).

## Results

**Figure 1:**
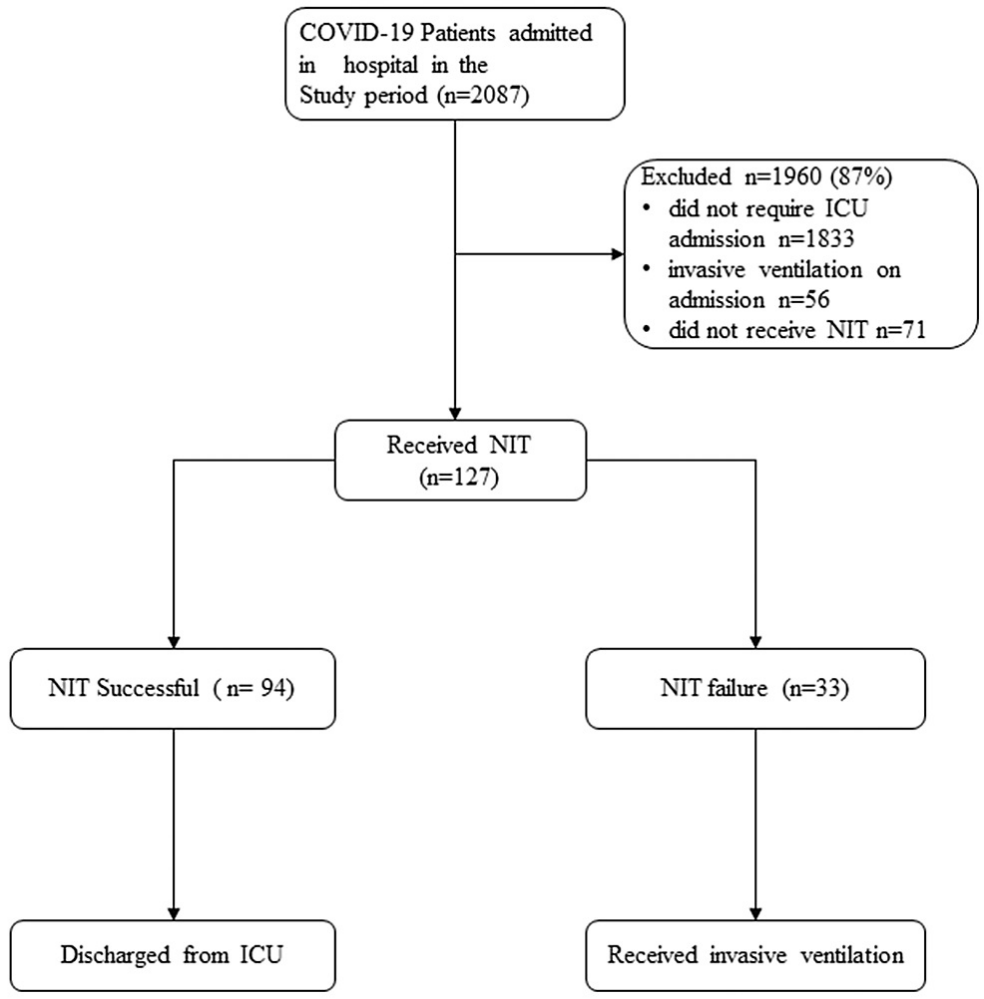
Participant flow COVID-19 - Coronavirus disease 2019, NIT - Non-invasive high flow oxygen therapy, ICU - Intensive care unit

During the study period, 2087 COVID- 19 patients were admitted to our hospital. Out of 254 patients admitted to ICU, 127 patients were initiated on NIT at admission, and they were included in the study (Figure 1). During the course of the ICU stay, 33 (26%) patients ultimately required invasive ventilation (NIT failure).

Demographic characteristics and clinical symptomatology are summarized in Table 1. The median age in NIT success group was 54 years (IQR 45-64) and 58 years (IQR 50-67) in NIT failure group; the male gender was predominant in both the groups (>60%). Fever and shortness of breath were the common symptoms in both groups. Frequent comorbidities in each group were hypertension, diabetes mellitus followed by obesity (BMI > 32 kg/m^2^). 92% (n=117) of the patients were on non-rebreathing mask with reservoir bag (15L/min oxygen) and the remaining patients were on venturi mask (FiO_2_=60%, O_2_ flow 15L/min) before initiation of NIT.

**Table 1:** Demographic characteristics and clinical profile of the study population NIT - Non-invasive oxygen therapy, HTN - hypertension, DM - diabetes, CAD - coronary artery disease, COPD - chronic obstructive pulmonary disease, BA - bronchial asthma, PTB - pulmonary tuberculosis, CKD - chronic kidney disease, CVA - cerebrovascular accidents, obesity (BMI ≥ 27kg/m^2^). *p-value < 0.05 is significant

Parameter	NIT success group (n=94)	NIT failure group (n=33)	P-value^*^
Demographics			
Age(y) (Median, IQR)	54 (45-64)	58 (50-67)	0.281
Male sex (n, %)	61 (65%)	20 (60%)	0.659
Symptoms			
Fever (n, %)	64 (68%)	22 (67%)	0.881
Dyspnea	86 (91%)	31 (94%)	0.653
Sore throat	10 (11%)	3 (9%)	0.801
Cough	20 (21%)	7 (21%)	0.994
Myalgia	7 (7%)	0	0.107
Diarrhea	6 (6%)	0	0.137
Comorbidities			
HTN	38 (40%)	18 (55%)	0.160
DM	40 (43%)	15 (45%)	0.772
CAD	9 (10%)	5 (15%)	0.379
Hypothyroid	9 (10%)	1 (3%)	0.452
COPD/BA	3 (3%)	0	0.567
PTB (old and active)	4 (4%)	1 (3%)	1.000
CKD	5 (5%)	5 (15%)	0.125
CVA	2 (2%)	0	1.000
Obesity	19 (20%)	9 (27%)	0.400

Procalcitonin, NT-proBNP, ferritin, SOFA score, LDH, and median duration of ICU stay was significantly high in the NIT failure group (p<0.05). PaO_2_/FiO_2_ (PFR) at two hours and six hours and ROX index at two and six hours after initiation of NIT were significantly higher in NIT success group (Table 2).

**Table 2:** Baseline investigations, PFR, SOFA score, ROX index and ICU length of stay in NIT success and NIT failure group NIT - Non-invasive Oxygen therapy, AST - aspartate transaminase, ALT - alanine transaminase, LDH - lactate dehydrogenase, CRP - C-reactive protein, NT -proBNP - N-terminal pro-B-type natriuretic peptide, PFR - PaO_2_/FiO_2_, SOFA - Sequential Organ Failure Assessment, ROX index - Ratio of Oxygen saturation index. *p-value < 0.05 is significant

Parameter	NIT success (n=94) Median (IQR)	NIT failure (n=33) Median (IQR)	P-value^*^
Hemoglobin (gm/dL)	11.85 (10.5-12.92)	11.65 (9.5-12.67)	0.739
White cell count (x10^9^)	10.5 (7.3-13.93)	12.4 (7.7-17.5)	0.069
Platelet Count (x10^9^/L)	228 (173-315)	219 (167-293)	0.310
Creatinine (mg/dl)	0.81 (0.68-0.97)	0.9 (0.74-1.08)	0.097
Bilirubin (mg/dl)	0.48 (0.38-0.68)	0.41 (0.31-0.67)	0.969
Albumin (g/dl)	3.28 (3.06-3.55)	3.27 (2.975-3.49)	0.508
AST (U/L)	46 (32-76)	50.6 (29.7-71.25)	0.456
ALT (U/L)	47.5 (29.07-73)	38.6 (33.25-67.1)	0.838
LDH (U/L)	461 (356-582)	615 (510-715)	0.002
CRP (mg/L)	91.96 (51.1-181)	97.24 (55.33-171.75)	0.919
Procalcitonin (ng/ml)	0.172 (0.049-0.376)	0.317 (0.148-0.907)	0.017
NT-Pro BNP (pg/ml)	341 (153.3-804)	499 (217.55-2439.75)	0.036
D Dimer (ng/ml)	861 (492-1867)	1153.5 (792-2512)	0.045
Ferritin (ng/ml)	673 (339-1182.5)	1069 (776-1402)	0.025
Fibrinogen (g/L)	6.71 (5.25-7.95)	6.54 (5.92-7.96)	0.638
PFR at 2 hours	109 (83-157)	80 (65-109)	0.007
PFR at 6 hours	125 (93-180)	103 (73-121)	0.017
SOFA score	6 (5-7)	7 (6-10)	<0.001
ROX 2 hours	3.54 (3.02- 3.83)	3.22 (2.87- 3.52)	0.025
ROX 6 hours	4.21 (3.84- 4.29)	3.85 (3.42- 4.22)	0.027
ICU length of stay (days)	6 (4-9)	15 (11-20)	0.001

Using univariate and multivariate logistic regression analysis, the possible predictors for NIT failure were identified and are listed in Table 3.

**Table 3:** Logistic regression analysis of possible predictors for NIT failure PFR - PaO_2_/FiO_2_, SOFA score - Sequential Organ Failure Assessment score, WBC - White Blood Cell, LDH - Lactate Dehydrogenase, CRP - C-reactive protein, NT-proBNP - N-terminal pro-B-type natriuretic peptide, ROX index- Ratio of Oxygen saturation index. *p-value < 0.05 is significant

Parameter	Univariate analysis Odds ratio (95% CI)	P value	Multivariate analysis Odds ratio (95% CI)	P-value
Age	0.98 (0.95- 1.01)	0.314		
Male sex	1.20 (0.53- 2.71)	0.660		
PFR 2 hours	1.01 (1.00- 1.01)	0.023	1.01 (0.99- 1.02)	0.503
PFR 6 hours	1.00 (1.00- 1.01)	0.024	1.00 (0.98- 1.01)	0.784
WBC count	0.91 (0.84- 0.98)	0.017	0.98 (0.84- 1.13)	0.755
Fibrinogen	0.96 (0.81- 1.14)	0.646		
Urea	0.99 (0.98- 1.00)	0.019	1.00 (0.99- 1.03)	0.353
Creatinine	0.88 (0.71- 1.10)	0.275		
LDH	1.00 (0.99- 1.00)	0.006	1.00 (0.99- 1.00)	0.837
Albumin	1.15 (0.48- 2.79)	0.750		
NT-proBNP	1.00 (1.00- 1.00)	0.738		
Procalcitonin	0.83 (0.67- 1.03)	0.092		
SOFA score	0.70 (0.57- 0.86)	0.001	0.88 (0.62- 1.25)	0.480
ROX 2 hours	30.75 (10.52- 89.84)	<0.001	32.35 (7.51- 139.39)	<0.001
ROX 6 hours	23.7 (8.35- 67.2)	<0.001	28.43 (6.17- 131.10)	<0.001
Ferritin	1.00 (0.99- 1.00)	0.268		
D-dimer	1.00 (1.00- 1.00)	0.909		
CRP	1.00 (0.98- 1.01)	0.628		
Obesity	0.68 (0.27- 1.70)	0.402		

**Figure 2:**
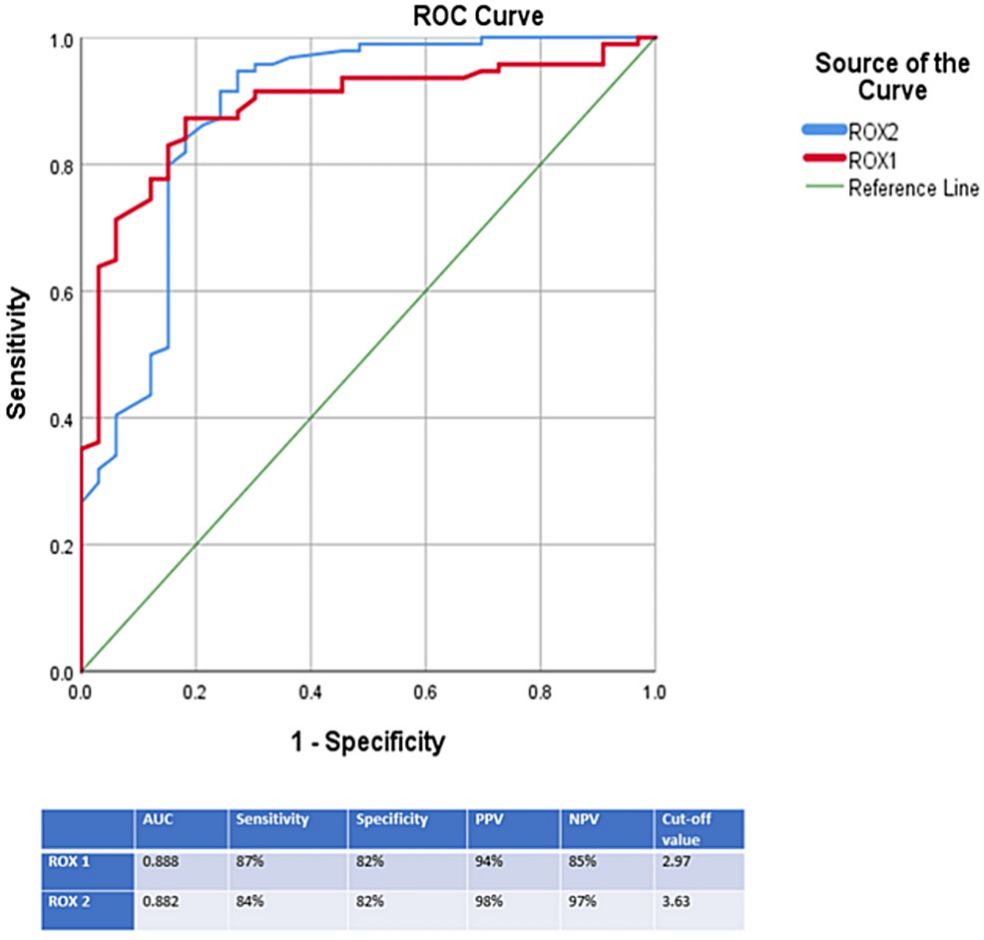
ROC curve analysis for predicting NIT failure AUC - Area under the curve, NPV - Negative predictive value, PPV - Positive predictive value

The multivariate model consisted of PFR at two and six hours, WBC count, urea, LDH, ROX index at two hours and six hours. ROX index at two hours and six hours remained significant in predicting NIT failure. Receiver operating characteristic curves were constructed for ROX index at two hours and ROX index at six hours using SPSS 24.0 for determining maximum sensitivity and specificity for predicting NIT failure. For ROX index at two hours, a cut-off of 2.97 below which it has a sensitivity of 87% and a specificity of 82% (positive predictive value 94%, negative predictive value 85%, area under the curve 0.888) for predicting NIT failure. The sensitivity of ROX index at six hours is increased compared to ROX index at two hours (92%) (cut-off value 3.63, positive predictive value 98%, negative predictive value 97%, AUC-0.896) (Figure 2).

## Discussion

In this retrospective study, the clinical characteristics of COVID-19 patients admitted to ICU were analyzed to identify the possible risk factors associated with NIT failure. About 26% of the patients failed to show improvement in NIT and subsequently received invasive ventilation. This NIT failure rate is similar to the previously published studies [7]. The majority of the patients (75%) in the present study received only HFNO therapy. The remaining patients received both CPAP NIV and HFNO. Lower ROX index at 2 and six hours were consistently associated with NIT failure. Early prediction of NIT failure helps in the institution of early elective invasive mechanical ventilation. Delaying mechanical ventilation in these patients would adversely affect the clinical outcome as evidenced by previous studies such as poor ventilator mechanics, increased mechanical ventilation duration, ICU stays, and complications of “crash” intubations [6,8].

The ROX index is defined as the ratio of SpO2/FiO2 (%) to respiratory rate (breaths/min). ROX index was initially reported by Roca et al. to predict HFNO success in patients with non-COVID-19 pneumonia [9]. Later on, the ROX index was validated in COVID-19 patients as a predictive tool for HFNO success [10-12]. The utility of the ROX index in predicting NIV CPAP failure in COVID-19 was studied by Yousuf et al. [13]. In our study, we have combined both HFNO and CPAP as NIT. NIT provides high flow, and the work of breathing is significantly reduced [14]. In a retrospective analysis of 324 patients with COVID-19 acute hypoxemic respiratory failure, Xu et al. reported that age ≥60 years, platelet count < 125x109/L, interleukin 6 > 7.0 pg/mL measured at initiation of HFNO, and ROX index of < 5.31 within four hours of HFNO initiation predicted HFNO failure [15]. In a smaller group of patients (n=40), Panadero et al. found that a ROX index < 4.94 measured after two to six hours of therapy predicted the need for endotracheal intubation [16]. In contrast to this, only a ROX index of < 2.97 at two hours and < 3.63 at six hours after NIT initiation predicted NIT failure in our study population. We also found that the sensitivity of predicting NIT failure was increased with six hours ROX index compared to two hours ROX index. The lower threshold can be explained by the following reasons. All of our patients were relatively younger and were treated in a dedicated COVID-19 tertiary care ICU with close monitoring.

During the second wave of the COVID-19 pandemic, objective tools for the identification of NIT failure were needed to guide resources and staff allocation. ROX index measured within the first six hours following initiation of NIT, predicted NIT failure in our study population. ROX index can be easily calculated at the bedside and should be routinely included as a vital sign in the case record. In addition to the ROX index, the SOFA score was also found to be one of the predictors of HFNO failure which signifies the influence of the multisystem involvement by COVID-19 on the HFNO therapy success [17,18]. Though the SOFA score was significant in univariate analysis in predicting NIT failure in our study, it failed to show in multivariate logistic regression analysis. In contrast to a previous study [19], NT-proBNP and D-dimer did not predict NIT failure in our cohort of patients. This observation can be explained by the following reasons. Being the tertiary care referral center, we received the majority of patients with higher SOFA scores (multiorgan dysfunction) intubated before admission to ICU and hence a relatively low-risk profile of patients who received NIT in our ICU. Inherent complications of invasive mechanical ventilation should be kept in mind while instituting early invasive mechanical ventilation.

Our study has a few limitations. Firstly, this is a retrospective study. Secondly, the influence of NIT failure and delayed intubation on mortality was not studied. Thirdly, this study did not include patients who received NIT in various wards of our hospital. Fourthly, some patients received NIT for a variable duration initially in the peripheral hospitals and were shifted to our tertiary care hospital ICU. Management protocols vary widely among hospitals and could confound the results significantly. The variable duration of illness, medical therapy, and duration of NIT in these subsets of patients could have influenced the outcome of NIT. Furthermore, the study population is small and monocentric.

## Conclusions

NIT is an excellent tool in the management of moderate to severe disease COVID-19 ARDS. However, patient selection is crucial for NIT success. In this retrospective study of 127 ICU patients with moderate to severe COVID-19 ARDS, 26% of patients received invasive mechanical ventilation following NIT. ROX index less than 2.97 at the second hour and ROX index less than 3.63 at the sixth hour of ICU admission were associated with endotracheal intubation. While SOFA score, PFR, and inflammatory markers did not predict NIT failure in our study cohort.

## Figures and Tables

**Figure 3:**
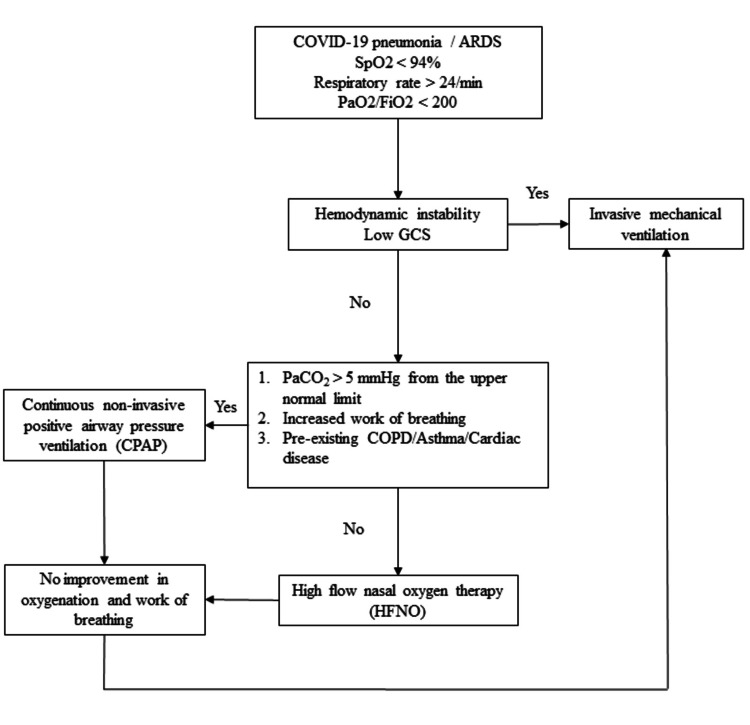
Oxygen therapy protocol for the management of moderate to severe COVID-19 in our hospital COVID-19 - Coronavirus disease 2019, ARDS - Adult respiratory distress syndrome, GCS - Glasgow coma scale, COPD - Chronic obstructive pulmonary disease
